# Effects of continuous positive airway pressure treatment on sleep architecture in adults with obstructive sleep apnea and type 2 diabetes

**DOI:** 10.3389/fnhum.2022.924069

**Published:** 2022-09-08

**Authors:** Kristine A. Wilckens, Bomin Jeon, Jonna L. Morris, Daniel J. Buysse, Eileen R. Chasens

**Affiliations:** ^1^Department of Psychiatry, School of Medicine, University of Pittsburgh, Pittsburgh, PA, United States; ^2^School of Nursing, University of Pittsburgh, Pittsburgh, PA, United States

**Keywords:** sleep, sleep apnea, diabetes, electroencephalography (EEG), spindles

## Abstract

Obstructive sleep apnea (OSA) severely impacts sleep and has long-term health consequences. Treating sleep apnea with continuous positive airway pressure (CPAP) not only relieves obstructed breathing, but also improves sleep. CPAP improves sleep by reducing apnea-induced awakenings. CPAP may also improve sleep by enhancing features of sleep architecture assessed with electroencephalography (EEG) that maximize sleep depth and neuronal homeostasis, such as the slow oscillation and spindle EEG activity, and by reducing neurophysiological arousal during sleep (i.e., beta EEG activity). We examined cross-sectional differences in quantitative EEG characteristics of sleep, assessed with power spectral analysis, in 29 adults with type 2 diabetes treated with CPAP and 24 adults undergoing SHAM CPAP treatment (total *n* = 53). We then examined changes in spectral characteristics of sleep as the SHAM group crossed over to active CPAP treatment (*n* = 19). Polysomnography (PSG) from the CPAP titration night was used for the current analyses. Analyses focused on EEG frequencies associated with sleep maintenance and arousal. These included the slow oscillation (0.5–1 Hz), sigma activity (12–16 Hz, spindle activity), and beta activity (16–20 Hz) in F3, F4, C3, and C4 EEG channels. Whole night non-rapid eye movement (NREM) sleep and the first period of NREM spectral activity were examined. Age and sex were included as covariates. There were no group differences between CPAP and SHAM in spectral characteristics of sleep architecture. However, SHAM cross-over to active CPAP was associated with an increase in relative 12–16 Hz sigma activity across the whole night and a decrease in average beta activity across the whole night. Relative slow oscillation power within the first NREM period decreased with CPAP, particularly for frontal channels. Sigma and beta activity effects did not differ by channel. These findings suggest that CPAP may preferentially enhance spindle activity and mitigate neurophysiological arousal. These findings inform the neurophysiological mechanisms of improved sleep with CPAP and the utility of quantitative EEG measures of sleep as a treatment probe of improvements in neurological and physical health with CPAP.

## Introduction

Obstructive sleep apnea (OSA) is defined as chronic, repeated reduction in airflow due to upper airway collapse or narrowing during sleep (Punjabi, 2008; Vijayan, 2012). Sleep apnea severely disrupts sleep (Verma et al., 2001) and has long-term health consequences (Punjabi, 2008; Vijayan, 2012). Treatments for sleep apnea such as continuous positive airway pressure (CPAP) significantly improve symptoms of OSA (Gay et al., 2006; Weaver et al., 2007) and typically lead to improvements in subjective sleep quality (Saunamäki et al., 2017) and reduced daytime sleepiness (Weaver et al., 2007). Studies of CPAP treatment have consistently demonstrated reduced time spent in lighter stages of sleep (Stage N1) and increased time spent in rapid eye movement (REM) sleep (Verma et al., 2001; Brillante et al., 2012) with treatment. However, there is contradictory evidence as to how CPAP influences stages of non-rapid eye movement (NREM) sleep, Stage N2 sleep and Stage N3 or slow-wave sleep (Issa and Sullivan, 1986; Verma et al., 2001; Djonlagic et al., 2021). These discrepancies make it challenging to determine the sleep-specific mechanisms and moderators of functional improvements with CPAP treatment.

Stages N2 and N3 are a relatively global reflection of the discrete neurophysiological sleep oscillations reflected in quantitative electroencephalography (EEG) power spectrum of sleep (Sprecher et al., 2015). Sleep oscillations, such as slow oscillation activity (0.5–1 Hz) and spindle activity (12–16 Hz), are neurophysiological oscillations involved in maintaining sleep and restoring cellular homeostasis (Tononi, 2009) as well as physiological, metabolic, and cognitive function (Mander et al., 2013; Staresina et al., 2015; Wilckens et al., 2016). Neurological health outcomes linked to these sleep oscillations include cognitive function (Anderson and Horne, 2003; Wilckens et al., 2016; Diep et al., 2020, 2021), memory consolidation (Staresina et al., 2015), and neurodegeneration (Winer et al., 2019; Mullins et al., 2020). Moreover, one’s ability to generate these sleep oscillations and concurrently minimize levels of neurophysiological arousal, indexed by beta activity (16–20 Hz) (Perlis et al., 2001; Hogan et al., 2020) is critical for initiating and maintaining a continuous and sound sleep (Besset et al., 1998; Perlis et al., 2001; Fang et al., 2017). Thus, examination of quantitative EEG spectral characteristics of sleep within these distinct frequency bands is important for understanding the mechanisms of improvements in sleep and function with CPAP treatment.

The slow oscillation predominates during Stage N3 sleep and involves globally synchronized synaptic activity (Amzica and Steriade, 1995; Neske, 2016). It reflects the interplay of cortical neurons with subcortical neurons of the thalamus, as well as the striatum and the cerebellum (Steriade et al., 1993; Roš et al., 2009; Neske, 2016). The slow oscillation is characterized by an “up-state,” reflecting brief periods of intense synaptic activity, and a “down-state” reflecting relative silence (Neske, 2016). The slow oscillation is considered to be among the most restorative aspects of NREM sleep, due to its role in biochemical maintenance of neurons, synaptic downscaling (Tononi, 2009), driving synchronization of other sleep oscillations (Steriade et al., 1993; Roš et al., 2009; Neske, 2016), consolidation of memories (Neske, 2016), as well as metabolic and immune functions (Tasali et al., 2008; Grimaldi et al., 2019). Moreover, greater functional and physiological significance is attributed to the slow oscillation, relative to higher frequency slow-wave activity (i.e., 1–4 Hz delta activity) (Mander et al., 2015; Winer et al., 2020). Lower levels of slow oscillation activity are associated with clinical, neurobehavioral, and neurological outcomes including disordered sleep (insomnia) (Hogan et al., 2020), lower prefrontal gray matter volume (Mander et al., 2013), older age (Mander et al., 2013), greater brain pathology (Mander et al., 2015), and lower cognitive abilities (Tononi, 2009; Mander et al., 2013). While a small number of studies have shown discrepant findings examining effects of CPAP on slow-wave activity, broadly defined (Gaudreau et al., 2001; Parekh et al., 2019), the slow oscillation has not been extensively examined in the context of sleep apnea and its treatment (Mullins et al., 2020).

Spindle activity is a hallmark of Stage N2 sleep and reflects bursts of oscillatory activity between 12–16 Hz (Schönauer and Pöhlchen, 2018). Spindles are generated by coordinated activity between the thalamus, the thalamic reticular nucleus, and the neocortex (thalamocortical loops) (Schönauer and Pöhlchen, 2018). Spindle activity prevents arousing stimuli from reaching the cortex (Saunamäki et al., 2017), and thereby prevents awakenings during NREM sleep. Greater spindle activity is associated with better cognitive abilities (Fogel and Smith, 2011; Fang et al., 2019) and facilitates the transfer of long-term memories from the medial temporal lobe to the cortex (Staresina et al., 2015). Spindle activity is commonly reduced in sleep apnea (Mullins et al., 2020; Muñoz-Torres et al., 2020). A small number of studies have demonstrated significant increases in spindle activity with CPAP (Chokroverty et al., 2015; Saunamäki et al., 2017; Yetkin and Aydogan, 2018).

Beta power predominates during wakefulness (Perlis et al., 2001) and is therefore considered a marker of neurophysiological arousal when elevated during sleep (Perlis et al., 2001; Hogan et al., 2020). Elevated beta power around sleep onset can increase sleep latency (Perlis et al., 2001) and leads to unwanted awakenings (Perlis et al., 2001). Beta power often has a reciprocal relationship with slow-wave activity (Perlis et al., 2001), potentially reflecting interference of high levels of beta activity with the restorative processes of slow-wave activity. Accordingly, elevated beta power is commonly found in patients with insomnia (Perlis et al., 2001; Hogan et al., 2020). However, little research has examined how sleep apnea and CPAP treatment influence beta power during sleep (Muñoz-Torres et al., 2020), despite the relevance of beta activity to initiating and maintaining sleep.

Finally, effects of sleep apnea on sleep architecture may depend on patient characteristics. For instance, effects may be greater and or unique in individuals with comorbid health conditions that affect sleep architecture. Type 2 diabetes (T2D) is associated with high prevalence of sleep apnea (Khandelwal et al., 2017) as well as disruptions to sleep architecture, including slow-wave sleep (Ahn et al., 2021), which may exacerbate metabolic complications (Chasens et al., 2013; Morris et al., 2018). These additional disruptions to sleep architecture may influence the degree to which CPAP treatment may improve sleep architecture in these individuals. Nonetheless, very little research has been conducted on the effects of sleep apnea treatment on sleep in T2D.

The current secondary analysis aimed to assess whether reduced AHI with CPAP titration is associated with differences in NREM sleep oscillations using quantitative EEG. We examined the association between CPAP treatment and key sleep oscillations [slow oscillation, sigma (spindle), and beta activity] in adults with OSA and T2D. Group differences in polysomnographic and spectral variables were tested among participants undergoing a CPAP treatment titration night and participants undergoing SHAM CPAP titration. Within-subject changes in polysomnographic and spectral variables were then examined before and after participants in the SHAM group crossed over to CPAP treatment at a second time point. We hypothesized that that after controlling for age and sex, CPAP treatment would be associated with greater slow oscillation power and spindle activity, and reduced beta activity, reflecting a higher arousal threshold and deeper, more restorative sleep with CPAP usage. This investigation may inform the mechanisms and moderators of functional improvements with CPAP, including physiological, metabolic, neurological, and cognitive function.

## Materials and methods

### Study overview

The current investigation was a secondary analysis from a clinical trial examining the effects of CPAP on glycemic control and diabetes management in individuals with T2D. The study involved two groups of participants: an active CPAP group and a SHAM CPAP group. Both groups completed overnight CPAP/SHAM titration studies including polysomnography (PSG), followed by 12 weeks of either active CPAP or SHAM. The SHAM CPAP group then crossed over to active CPAP and participated in a second overnight titration sleep study before beginning 12 weeks of active CPAP. The PSG data from the CPAP/SHAM titration night was used for the current analyses. The current investigation focused on effects of CPAP treatment on PSG-assessed sleep and quantitative EEG. This study was approved by the University of Pittsburgh Internal Review Board.

### Parent study

The Diabetes Sleep Treatment Trial (DSTT), was a randomized clinical trial designed to examine the effect of CPAP treatment on glycemic control and self-management behavior in persons with T2D. Details of the scientific premise, design, and methodology of the DSTT are published elsewhere (Chasens et al., 2019). In brief, potential participants with self-reported T2D and symptoms of OSA (snoring or breath holding) or “poor sleep quality” were recruited from the community, diabetes clinics, and sleep clinics. Inclusion and exclusion criteria have been published (Chasens et al., 2019). Briefly, inclusion criteria included an apnea hypopnea index (AHI) ≥ 10 events per hour on a home sleep study with an ApneaLink Plus^®^, 18 years of age or older, and T2D mellitus. Exclusion criteria included HbA1c levels < 6.5% or > 11%, acute medical or surgical conditions or hospitalization within the past 3 months, not CPAP naïve or previous treatment of sleep apnea, prior diagnosis of another sleep disorder, history of a car crash secondary to sleepiness, and work in a safety sensitive occupation. OSA severity was not otherwise considered for eligibility purposes.

The baseline assessment included body mass index and glycemic control (HbA1C). Questionnaires included self-reported sleep history (diagnosis of insomnia, restless leg syndrome, or OSA), the Insomnia Severity Index (Bastien et al., 2001), Epworth Sleepiness Scale (Johns, 1991), and Pittsburgh Sleep Quality Index (Buysse et al., 1989). Participants enrolled in the study were randomized to either SHAM or CPAP and underwent a single laboratory titration study. After 12-weeks of treatment, participants were unblinded and those originally on SHAM were encouraged to have a second titration to therapeutic CPAP.

### Participants

Data from in-lab PSG studies on CPAP/SHAM titration nights were analyzed. Participants were ages 35–80 (Chasens et al., 2019). Sixty-three participants out of 67 had usable PSG data. Demographic and clinical data are presented in Table 1 across participants with available PSG data. Participants with less than 4 h of total sleep time during the PSG night were excluded from analyses with spectral variables. This cutoff excluded participants with less than 2 NREM cycles and ensured that a sufficient amount of data contributed to spectral variable averages to mitigate confounds of differences in total sleep time when assessing group differences in spectral characteristics. After excluding participants with total sleep time < 4 h or significant EEG artifact, 53 participants had usable spectral data at T1 (29 active CPAP, 24 SHAM CPAP). There were 27 participants included in the within-subjects analysis of time point (24 T1 SHAM and 19 T2 CPAP cross-over). The sample for the within-subjects analysis of time point had 3 participants with usable T2 PSG data, but unusable T1 PSG data.

**TABLE 1:** Demographic and clinical characteristics in participants undergoing active CPAP and participants undergoing SHAM CPAP.

	Active CPAP	SHAM	Group differences *F*(1, 57)/chi^2^	Group differences p
N	32	31		
Age mean (SD)	61.19 (9.8)	55.71 (9.07)	5.29	0.025
Sex (% female)	62.5%	48.4%	1.3	0.260
Race (% white)	71.9%	77.4%	0.26	0.613
Education (years)	14.94 (3.27)	15.0 (3.19)	0.38	0.541
BMI	35.73 (6.45)	37.23 (7.62)	0.22	0.639
HbA1c	7.67 (0.73)	8.04 (1.1)	0.39	0.535
ISI total	13.81 (5.68)	13.45 (5.65)	0.01	0.915
Epworth	10.16 (3.98)	9.48 (5.06)	0.66	0.421
PSQI total	9.91 (3.89)	9.90 (4.00)	0.002	0.969
AHI	20.73 (9.65)	22.73 (14.05)	0.39	0.534
RI	23.53 (9.59)	25.51 (13.68)	0.33	0.568

Means (SD) reflect samples with participants with available PSG data. ISI is missing data from 2 participants. F and p statistics reflect between groups MANCOVA with all participants with PSG data controlling for age and sex. A separate ANOVA was run for age. Chi^2^ and p statistics reflect separate chi^2^ tests for sex and race. BMI, body mass index (kg/m^2^); HbA1C, glycated hemoglobin; ISI, Insomnia Severity Index; PSQI, Pittsburgh Sleep Quality Index; AHI, apnea + hypopnea index; RI, respiratory index. AHI and RI reflect the screening ApneaLink^®^ Plus scores.

### Active and SHAM continuous positive airway pressure intervention

Active and SHAM CPAP titration studies had identical protocols but only participants in the active group were titrated with positive pressure to reduce AHI to < 5 or the best AHI achievable at a CPAP pressure that was tolerated. Gentle airflow in the SHAM CPAP units was used to prevent CO_2_ retention and to mimic active CPAP with positive pressure less than 1 cm. The morning after the titration study, participants took home active CPAP or SHAM CPAP machines that looked identical. The SHAM machines had a resister built in and air vents to give the feel of active CPAP but without therapeutic positive pressure.

### Polysomnography data collection, scoring, and spectral analysis

Laboratory-based PSG was recorded on the titration night for the CPAP and SHAM group, and then again on the cross-over titration night for the SHAM group. The titration procedure was performed using standard techniques (Smith et al., 1995) with an effective CPAP level of AHI < 5 per hour, which has been described previously (Chasens et al., 2019). PSG took place at the participants’ habitual sleep times as assessed by sleep diary. EEG data was recorded by Grass Telefactor Model 15 Amplifier to Harmonie Stellate/Natus Systems. EEG channels acquired were F3, F4, C3, C4, O1, and O2 referenced to A1–A2. Signal filters were 0.3 Hz high-pass and 100 Hz low-pass with 60 Hz notch filter. Signal was bandlimited by 4-Pole Bessel anti-aliasing filter. EEG signals were digitized at a rate of 256 Hz. Raw digitized data were band-limited using a 60 Hz low-pass finite impulse response filter, then decimated to 128 Hz for quantitative analysis.

The sleep record was visually scored in 30-s epochs using American Academy of Sleep Medicine criteria (Iber Sa-I and Quan, 2007). Four-second epochs identified as movement artifact by an automated algorithm (Brunner et al., 1996) and visual inspection were excluded from the record. The spectral analysis was performed on frontal (F) and central (C) channels with a 512-point fast Fourier transform using epochs scored as NREM sleep as well as epochs scored as REM sleep (Vasko et al., 1997). The complete description of the algorithm used for spectral analysis is published elsewhere (Vasko et al., 1997). Briefly, the EEG was first segmented into 0.25 Hz bins over 4-s epochs, which allows for power spectral analysis with a frequency resolution of 1/4 Hz. Then each segment was weighted by a Hamming window and the modified periodogram was computed. The modified periodogram is the magnitude-squared of the discrete Fourier transform of the weighted segment. The resulting discrete spectrum was arranged into frequency bands, each with an associated power spectral density of the Hamming windowed EEG (in units of μV^2^/Hz) (Vasko et al., 1997). Within-band power for all of NREM sleep and for each NREM period was calculated for F3, F4, C3, and C4 channels beginning after sleep onset.

Following the Fourier transform, power was averaged across 0.5 Hz bins from 0.5 to 32 Hz. These values were summed across 0.5 Hz bins to obtain total power (area under the curve) across frequency bands and within each frequency band. To calculate average power, total power within a frequency band was divided by the number of 0.5 Hz bins within each frequency band of interest [0.5–1 Hz (slow oscillation), 12–16 Hz (sigma/spindle activity), and 16–20 Hz (beta activity)]. Relative power was calculated for each frequency band by dividing total power within each frequency band of interest by total power across the entire 0.5–32 Hz range.

We included both average power and relative power for the current analysis. By examining both average and relative power, we were able to assess sleep oscillation power in absolute terms as well as power relative to total EEG power across the 0.5–32 Hz range, which varies between individuals. We tested both F and C channels to explore whether effects involving the slow oscillation were greater over F channels where the slow oscillation predominates, and whether effects involving spindle activity were greater over C channels where spindle activity predominates. Analyses limited to the first NREM period were examined to assess the period in which the greatest homeostatic sleep effects would be observed.

### Statistical analyses

Linear mixed models tested group differences in PSG variables and spectral variables as well as change in PSG and spectral variables for SHAM to CPAP cross-over. PSG variables were sleep latency, total sleep time, sleep efficiency, wake after sleep onset, percent and minutes of each sleep stage (N1, N2, N3, REM), and REM latency. Spectral variables assessed were average and relative slow oscillation, sigma, and beta power. Compound symmetry was selected as the repeated covariance structure for change analyses with SHAM to CPAP cross-over. Maximum likelihood was selected as the estimation method. Mixed models were selected to include all data available in analyses regardless of missingness across time points and EEG channels. Age and sex were included as covariates in all mixed model analyses.

For mixed models testing group differences, participant number was a random effect. Channel (F, C) was included as a repeated measure, group was a fixed factor, and age and sex were covariates. Each PSG and spectral variable was included as the dependent variable in separate models.

For mixed models testing change from SHAM to CPAP, participant number was included as a random effect. Time point (pre, post) and channel (F, C) were included as a repeated measure. Group was a fixed factor and age and sex were covariates. Each PSG and spectral variable were included as the dependent variable in separate models.

Due to the limited sample size to test and detect interactions, we did not formally test interactions as a function of NREM period or channel. Instead, we conducted mixed model analyses first across whole night NREM sleep, then again within the first NREM period separately. To test whether effects differed for F and C channels, we followed up significant effects by testing them separately for F channels and C channels. Results for all frequency bands from 0.5 to 20 Hz are presented in the Supplementary Material section.

## Results

### Group differences and within-subjects effects of continuous positive airway pressure on polysomnography variables

Table 2 displays effects of group (active and SHAM) and time point (SHAM to active CPAP cross-over) on PSG variables. Analyses with group revealed that the active CPAP group had significantly greater sleep efficiency and percent rapid eye movement (REM) sleep, as well as lower WASO, percent Stage N1 and shorter REM latency. No other group differences were found in PSG variables.

**TABLE 2:** PSG data for active and SHAM CPAP, and after cross-over to CPAP within the SHAM group.

	Active CPAP (SD)	SHAM T1 (SD)	Group difference F	Group difference p	SHAM to CPAP T2 (SD)	Change F	Change *p*
N PSG data	32	31			19		
N Spectral data	29	24			18		
Sleep latency (min)	24.61 (30.2)	29.79 (35.9)	0.53	0.471	**13.55 (10.8)**	**4.63**	**0.043**
Total sleep time (min)	351.59 (73.2)	334.27 (137.7)	1.26	0.265	380.21 (101.3)	1.85	0.195
**Sleep efficiency**	**74.75 (12.4)**	**65.16 (25.5)**	**5.69**	**0.02**	**77.63 (15.7)**	**4.45**	**0.047**
**WASO (min)**	**92.58 (48.4)**	**130.87 (84.8)**	**8.43**	**0.005**	**86.74 (49.7)**	**4.73**	**0.042**
Stage N1 (min)	36.63 (19.8)	40.05 (28.0)	0.67	0.416	38.29 (24.1)	0.08	0.778
**Percent stage N1**	**11.23 (8.2)**	**18.74 (20.4)**	**5.26**	**0.025**	10.53 (6.3)	3.30	0.083
Stage N2 (min)	205.41 (55.7)	205.71 (91.3)	0.15	0.703	236.47 (74.4)	1.35	0.263
Percent stage N2	58.14 (8.0)	58.49 (15.0)	0.01	0.942	61.71 (9.6)	0.13	0.720
Stage N3 (min)	26.27 (33.8)	21.60 (35.1)	1.09	0.300	20.76 (24.0)	0.32	0.580
Percent stage N3	7.55 (10.0)	5.32 (7.9)	2.39	0.127	6.95 (9.2)	1.26	0.277
**Percent REM**	**23.07 (8.9)**	**17.45 (9.3)**	**6.51**	**0.013**	20.81 (9.0)	1.72	0.201
**RLA (*n* = 32, 28, 18)**	**85.16 (52.1)**	**112.64 (57.8)**	**5.57**	**0.022**	106.22 (70.0)	0.29	0.597

Raw means (SD) for PSG variables across all participants with available PSG data. Bold font denotes significance, p < 0.05.

SHAM to CPAP cross-over analyses revealed significant improvements in sleep latency, sleep efficiency, and WASO. No sleep stages showed significant change effects with SHAM-CPAP cross-over.

### Group differences in spectral variables

Table 3 displays group differences in spectral variables. There were no group differences in any spectral measures, slow oscillation power, sigma power, or beta power. There were also no group differences when analyses were limited to the first NREM period (Supplementary Table 3).

**TABLE 3:** Between group differences for active CPAP and SHAM CPAP at Time Point 1; Results are presented across the whole night.

Between subject main effects of group *N* = 53								

	Average power				Relative power			
		
	F	Estimate (se)	df	*p*	F	Estimate (se)	df	*p*
**Slow**	0.01	−0.80 (7.74)	1, 53.40	0.918	1.54	−0.02 (0.12)	1, 52.94	0.221
**Sigma**	0.5	0.06 (0.09)	1, 53.08	0.484	0.15	−0.002 (0.004)	1, 53.16	0.697
**Beta**	0.001	−0.001 (0.02)	1, 52.92	0.978	0.34	−0.001 (0.001)	1, 58.13	0.565

Parameter estimates are for the effect of group with SHAM being the reference group. There were no between group differences in any spectral variables.

### Effects of SHAM to continuous positive airway pressure cross-over on spectral variables

**FIGURE 1:**
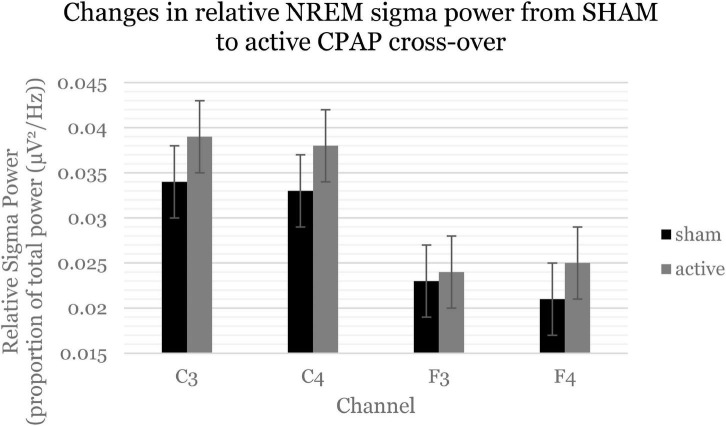
Sigma power (12–16 Hz, spindle activity) increased across the whole night and consistently across channels. Estimated marginal means and standard errors from the mixed model are displayed.

**FIGURE 2:**
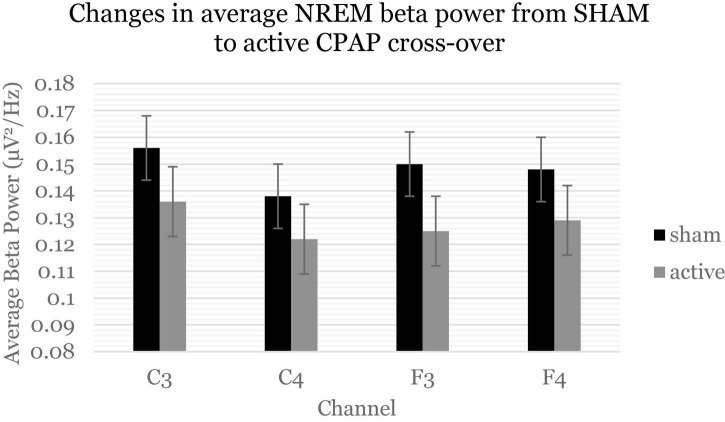
Beta power (16–20 Hz) decreased across the whole night and consistently across channels. Estimated marginal means and standard errors from the mixed model are displayed.

Table 4 displays change effects of SHAM to active CPAP cross-over on spectral variables. Across the whole night, there was a significant increase in sigma power (12–16 H) for relative power with cross-over from SHAM to CPAP (Table 4 and Figure 1). This effect was not significant for average power or for F and C channels analyzed separately, *F*(1, 59.21) = 2.7, *p* = 0.105; *F*(1, 58.75) = 2.6, *p* = 0.109. Average beta activity (16–20 Hz) decreased with CPAP across the whole night (Table 4 and Figure 2). This effect did not reach significance for relative power but was consistent for average power across F and C channels, *F*(1, 60.41) = 6.5, *p* = 0.014; *F*(1, 60.63) = 5.7, *p* = 0.020. There were no significant changes in whole night slow oscillation power.

**TABLE 4:** Sham to CPAP cross-over within subjects change effects for spectral variables from the mixed model.

	Average power				Relative power			
		
	*F*	Estimate (se)	df	*p*	*F*	Estimate (se)	df	*p*
**Slow oscillation**	2.06	4.1 (2.9)	1, 141.48	0.154	0.49	0.004 (0.006)	1, 138.70	0.484
**Sigma**	1.61	−0.027 (0.02)	1, 141.47	0.206	**4.82**	−**0.004 (0.002)**	**1, 142.45**	**0.030**
**Beta**	**10.2**	**0.019 (0.006)**	**1, 144.15**	**0.002**	2.62	0.0007 (0.0004)	1, 143.76	0.108

Parameter estimates are for timepoint with timepoint 2 being the reference (negative parameter estimates correspond to an increase with CPAP). Bold font denotes significance, p < 0.05.

**FIGURE 3:**
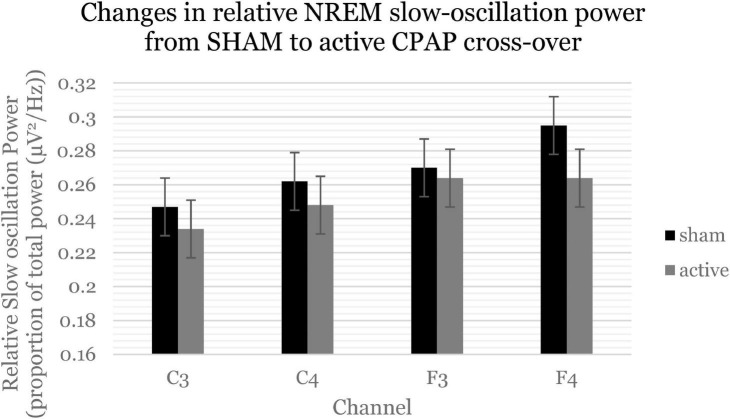
Slow oscillation power (0.5–1 Hz) significantly decreased within the first NREM period. Estimated marginal means and standard errors from the mixed model are displayed.

Within the first NREM period, there were significant decreases in slow oscillation power (0.5–1 Hz) across relative and average power (Figure 3 and Supplementary Table 4). Follow-up analyses showed that these effects were statistically significant for F channels [average *F*(1, 57.85) = 6.2, *p* = 0.016; relative *F*(1, 57.20) = 6.9, *p* = 0.011] but not C channels. Within the first NREM period, there was again a significant decrease in beta activity, which was not significant when F and C channels were analyzed separately. There were no significant changes in sigma activity specifically within the first NREM period.

## Discussion

Active CPAP treatment was associated with improved sleep efficiency, WASO, lower Stage N1 sleep, greater REM sleep, and lower REM latency. CPAP treatment was also associated with changes in key sleep oscillations: an increase in sigma activity and a decrease in beta activity across the whole night for the SHAM to CPAP cross-over study. Secondarily, the SHAM to CPAP cross-over study demonstrated decreases in the slow oscillation within the first NREM period. These results suggest within-subject improvements in sleep architecture in terms of increased spindle and decreased beta activity with CPAP, which may improve arousal threshold during NREM sleep. However, the unexpected decrease in the slow oscillation within the first NREM period suggests a significant reduction in one key feature of sleep architecture considered a marker of sleep depth and synaptic homeostasis.

### Group differences

Compared to SHAM, active CPAP was associated with several changes in PSG measures: reduced wake after sleep onset, a shorter REM latency, and greater percent REM sleep in the active CPAP group. These results are consistent with prior studies demonstrating an improvement in sleep and a rebound increase in REM sleep with CPAP treatment (Koo et al., 2012). A REM rebound with CPAP is common because the loss of muscle tone during REM sleep leads to high incidences of apneas (Ralls and Cutchen, 2019) that disrupt REM sleep. This leads to an increase REM sleep “pressure” (Koo et al., 2012). While we did not find significant improvements in slow-wave sleep, effects of CPAP on slow-wave sleep may be moderated by various factors. Slow-wave sleep has also shown inconsistent results in other studies of CPAP. Some studies have suggested that slow-wave sleep does not rebound to the same extent as REM sleep (Aldrich et al., 1989; Espinar Sierra, 1989; Osuna et al., 2008) and that improved sleep quality and compliance may be more attributable to a REM rebound (Koo et al., 2012). Sleep disordered breathing that occurs primarily during REM sleep has been shown to be greater among individuals with cardiovascular disease (Aurora et al., 2018). Thus, the influence of CPAP on slow-wave sleep may depend on how comorbidities and sleep apnea interact to affect sleep stages. Studies examining apneas as a function of sleep stage and T2D relative to control participants or patients with other comorbidities would be necessary to test this hypothesis.

In terms of spectral measures, there were no group differences in the slow oscillation, sigma activity, or beta activity. The lack of group differences in each of the spectral measures here may reflect stronger effects on REM sleep shown in the literature (Koo et al., 2012) or sleep continuity, more broadly. Alternatively, any groups differences as a function of CPAP may be washed out by baseline group differences prior to treatment which were not assessed here.

### Within subjects effects of continuous positive airway pressure

Across the whole night, cross-over from SHAM to active CPAP was associated with increased relative sigma power (12–16 Hz), providing evidence of improvements in EEG activity within the spindle range across the whole night with CPAP. Sleep spindles are thought to prevent arousing stimuli from being processed by the cortex and thereby prevent arousals during NREM sleep (Knoblauch et al., 2002; Peter-Derex et al., 2012). Such changes with CPAP may partially explain improved sleep continuity and sleep quality with CPAP usage (Quan et al., 2018). Future studies may examine how these improvements in spindle activity may lead to long-term positive consequences of sleep apnea treatment (Mullins et al., 2020), such as improvements in cognitive and brain health as well as improved function and health trajectories with CPAP usage (Mullins et al., 2020).

Higher spindle activity is commonly found in healthy controls compared with individuals with OSA (Mullins et al., 2020) and is consistent with the few preliminary studies that have examined effects of CPAP on spindles (Chokroverty et al., 2015; Yetkin and Aydogan, 2018). Indeed, one other study demonstrated partial normalization of spindle activity with one night of CPAP usage (Saunamäki et al., 2017). The current study did not demonstrate a baseline deficit in sigma (spindle) activity in the cross-sectional group comparison. Nonetheless, the increase in sigma activity could be driven by a variety of inter-related mechanisms, including homeostatic pressure for sigma activity, as sigma activity has also been shown to increase after sleep deprivation (Knoblauch et al., 2003). This increase in sigma activity may increase arousal threshold and consolidate sleep. Alternatively, more consolidated sleep due to reduced apneas may allow for greater opportunities for sigma activity.

The findings of reduced average beta activity similarly may contribute to an overall improvement in sleep efficiency found here and in other studies (Loredo et al., 2006). Lower levels of beta activity reflect lower neurophysiological arousal during sleep (Perlis et al., 2001). Reduced neurophysiological arousal may contribute to the observed reduction in sleep latency and increase in sleep efficiency. Alternatively, the reduction in beta activity may also be due to fewer awakenings, such that sleep immediately following an arousal from an apnea may be marked by higher levels of neurophysiological arousal, manifested as beta activity during NREM sleep.

We found an unexpected reduction in average and relative slow-wave activity within the first NREM period, particularly for F channels. A decrease in the slow oscillation could be interpreted to suggest that CPAP is detrimental for the slow oscillation. However, a decrease in the slow oscillation could result from relatively preserved slow oscillation power relative to REM sleep in the current sample. The homeostatic drive for other features of sleep, such as REM sleep (Datta et al., 2015), may be stronger if they are more persistently inhibited before treatment. The current slow oscillation findings, nonetheless, are informative for studies examining sleep-specific mechanisms of functional and physiological improvements with CPAP treatment. A reduction in the slow oscillation within the first NREM period, may reflect a reduction in processes that contribute to restorative and sound sleep, and functional outcomes including glucose homeostasis (Koren et al., 2011).

### Limitations and future directions

The current study should be interpreted in the context of the following limitations. The current sample size was relatively small and may have been insufficient to detect smaller magnitude effects sizes between groups. Inconsistencies in significance between measures of average and relative power for sigma and beta power were also likely due to the small sample size. A larger sample size would allow us to interpret whether results are specific to average or relative power and assess cross-frequency dynamics. The study design did not include a baseline PSG study to determine baseline levels of sleep architecture before active or SHAM CPAP. This study design was chosen to mimic standard clinical practice and to ensure that participants were not prevented from receiving treatment. However, the design prevents us from covarying for baseline power in each band, which may be important given substantial inter-individual variability. Such variability may also explain why we observed longitudinal within-subject effects, but not cross-sectional between-subject effects.

First night effects may have influenced the current findings. The first night effect is a well-established phenomenon (Agnew et al., 1966), whereby a participant’s sleep is poorer on the first night in the laboratory compared with subsequent nights. In the SHAM to active CPAP cross-over study, SHAM always occurred before active CPAP, which may have influenced change effects reported here. Nonetheless, the SHAM and active CPAP nights were 12 weeks apart, which may have washed out first night effects. Further, the improvement in sleep latency with cross-over may partially reflect a first night effect.

The slow oscillation findings from 0.5 to 1 Hz should be interpreted with caution due to likely signal attenuation at this range when using a 0.3 Hz high pass filter. Signal attenuation may be greater for frequency bands closer to this filter cut-off, such as the slow oscillation. Nonetheless, the level of signal attenuation would be expected to be similar between groups and within participants. Moreover, individual differences in when participants reached therapeutic pressure likely introduced variability in the magnitude of treatment on NREM sleep, particularly effects within the first NREM period.

While the current findings are informative for understanding the sleep-specific mechanisms of functional changes with CPAP, the current investigation did not examine the functional consequences of these changes in sleep architecture. Future work should investigate quantitative EEG sleep characteristics as a mediator or moderator of long-term changes in health and function with CPAP treatment.

## Conclusion

The current investigation demonstrated that CPAP treatment is associated with greater REM sleep, and within-subject improvements in spindle activity and neurophysiological arousal assessed with sigma and beta activity in individuals with T2D. These findings inform the neurophysiological mechanisms of improved sleep with CPAP and the utility of quantitative EEG measures of sleep oscillations as a treatment probe of functional improvements with CPAP. Future studies may examine how these sleep improvements lead to long-term effects on metabolic as well as neurological outcomes.

## Data availability statement

The raw data supporting the conclusions of this article will be made available by the authors, without undue reservation.

## Ethics statement

The studies involving human participants were reviewed and approved by the University of Pittsburgh Internal Review Board. The patients/participants provided their written informed consent to participate in this study.

## Author contributions

EC designed the study. KW, EC, and DB defined the research objective. KW performed the analyses with input from EC, BJ, and DB. KW wrote the manuscript with critical input from BJ, JM, DB, and EC. All authors contributed to the article and approved the submitted version.
