# Supplementary material for: Effects of continuous positive airway pressure treatment on sleep architecture in adults with obstructive sleep apnea and type 2 diabetes
Source: Front Hum Neurosci. 2022 Sep 8;16:924069. doi: 10.3389/fnhum.2022.924069 (PMC9513763; doi:10.3389/fnhum.2022.924069)
Supplement: Supplementary file 1 [file Data_Sheet_1.docx]

**Supplementary Materials**

Table of Contents:

1. Results for all frequency bands 0.5-20 Hz across the whole night
   1. Between group differences for all frequency bands 0.5 -20 Hz across the whole night (Table 1)
   2. Sham to CPAP cross-over effects for all frequency bands 0.5- 20 Hz across the whole night (Table 2)
2. Results for all frequency bands 0.5-20 Hz within the first NREM period
   1. Between group differences for all frequency bands 0.5-20 Hz within the first NREM period (Table 3)
   2. Sham to CPAP cross-over effects for all frequency bands 0.5-20 Hz within the first NREM period (Table 4)
3. **Results for all frequency bands .5-20 Hz across the whole night**

**a)**

| **Supplementary Table 1.** Between group differences for all frequency bands through 20 Hz across the whole night**.** | | | | | | | | |  | |
| --- | --- | --- | --- | --- | --- | --- | --- | --- | --- | --- |
|  | **Average Power** | | | | **Relative Power** | | |  | |  |
|  | F | Estimate (se) | df | P | F | Estimate (se) | Df | p | |  |
| **Slow** | .011 | -.80 (7.74) | 1,53.40 | .918 | 1.536 | -.02 (.12) | 1,52.94 | .221 | |  |
| **Delta** | .143 | 10.53 (27.83) | 1,53.49 | .707 | .015 | .012 (.1) | 1,53.07 | .903 | |  |
| **Theta** | .331 | .17 (.29) | 1,53.13 | .568 | .182 | .003 (.007) | 1,53.31 | .672 | |  |
| **Alpha** | .136 | .08 (.24) | 1,53.10 | .714 | .079 | .002 (.007) | 1,53.06 | .780 | |  |
| **Sigma** | .498 | .06 (.09) | 1,53.08 | .484 | .154 | -.002 (.004) | 1,53.16 | .697 | |  |
| **Beta** | .001 | -.001 (.02) | 1,52.92) | .978 | .336 | -.001 (.001) | 1,58.13 | .565 | |  |

**b)**

| **Supplementary Table 2.** Sham to CPAP cross-over effects for all frequency bands through 20 Hz across the whole night**.** | | | | | | | | |  | |
| --- | --- | --- | --- | --- | --- | --- | --- | --- | --- | --- |
|  | **Average Power** | | | | **Relative Power** | | |  | |  |
|  | F | Estimate (se) | df | P | F | Estimate (se) | Df | p | |  |
| **Slow** | 2.06 | 4.1 (2.9) | 1, 141.48 | .154 | .493 | .004 (.006) | 1,138.70 | .484 | |  |
| **Delta** | .865 | 7.98 (.857) | 1, 139.83 | .354 | .802 | .03 (.04) | 1,142.39 | .372 | |  |
| **Theta** | .839 | .06 (.07) | 1, 136.68 | .361 | .004 | -.0001 (.002) | 1,139.72 | .950 | |  |
| **Alpha** | .893 | .04 (.04) | 1, 138.25 | .346 | 1.396 | -.002 (.002) | 1,140.35 | .239 | |  |
| **Sigma** | 1.613 | -.027 (.02) | 1,141.47 | .206 | **4.817** | **-.004 (.002)** | **1,142.45** | **.030** | |  |
| **Beta** | **10.198** | **.019 (.006)** | **1,144.15** | **.002** | 2.622 | .0007 (.0004) | 1,143.76 | .108 | |  |

1. **Results for all frequency bands .5-20 Hz within the first NREM period**

**a)**

**Supplementary Table 3:** Between group differences for all frequency bands .5-20 Hz within the first NREM period

|  | | | | | |
| --- | --- | --- | --- | --- | --- |
|  |  | Average Power | | Relative Power | |
|  |  | F | P | F | p |
| **Slow** | **.5-1 Hz** | .481 | .491 | 2.741 | .104 |
| **Delta** | **1-4 Hz** | .040 | .842 | .585 | .448 |
| **Theta** | **4-8Hz** | .802 | .374 | 1.347 | .251 |
| **Alpha** | **8-12 Hz** | .424 | .518 | .791 | .378 |
| **Sigma** | **12-16 Hz** | .312 | .579 | .029 | .865 |
| **Beta** | **16-20 Hz** | .215 | .645 | .138 | .712 |

**b)**

**Supplementary Table 4:** Sham to CPAP cross-over effects for all frequency bands 0.5-20 Hz within the first NREM period**.**

|  | | | | | |
| --- | --- | --- | --- | --- | --- |
|  |  | Average Power | | Relative Power | |
|  |  | F | P | F | p |
| **Slow** | **.5-1 Hz** | **5.191** | **.024** | **7.349** | **.008** |
| **Delta** | **1-4 Hz** | .876 | .351 | .206 | .650 |
| **Theta** | **4-8Hz** | .068 | .795 | .686 | .409 |
| **Alpha** | **8-12 Hz** | .638 | .426 | 2.246 | .136 |
| **Sigma** | **12-16 Hz** | 1.779 | .184 | 2.159 | .144 |
| **Beta** | **16-20 Hz** | **5.368** | **.022** | **3.962** | **.048** |
